# A new high temperature, high heating rate, low axial gradient capillary heater

**DOI:** 10.1107/S1600577522009845

**Published:** 2023-01-01

**Authors:** Kenneth P. Marshall, Hermann Emerich, Charles J. McMonagle, Chloe A. Fuller, Vadim Dyadkin, Dmitry Chernyshov, Wouter van Beek

**Affiliations:** a Swiss–Norwegian Beamlines, European Synchrotron Radiation Facility, 71 Avenue des Martyrs, 38000 Grenoble, France; ESRF – The European Synchrotron, France

**Keywords:** capillary heater, X-ray scattering, X-ray absorption

## Abstract

A new heater design, capable of fast heating and cooling to and from >1000°C, has been developed at the Swiss–Norwegian Beamlines, European Synchrotron Radiation Facility, France. The design uses a SiC head to distribute heat, and resistive Si_3_N_4_ heat cartridges to provide heat.

## Abstract 2

A new high-temperature capillary heater design suitable for synchrotron X-ray diffraction, total scattering and EXAFS experiments is described.

## 1. Introduction

Temperature is an important thermodynamic field that defines the stability and functional behaviour of many materials. Temperature-dependent diffraction is one of the most powerful tools for characterizing solid materials, providing atomic-level detail on the mechanisms underlying structural phase transformations (Tomaszewski, 1992 ▸; Whatmore, 2017 ▸; Bassett & Brown, 1990 ▸) and chemical reactions (Bøjesen & Iversen, 2016 ▸; Epple, 1994 ▸; Bassett & Brown, 1990 ▸), and untangling different contributions in the thermal expansion coefficients (James *et al.*, 2001 ▸; Bassett & Brown, 1990 ▸) to name a few. Nowadays, measurements as a function of temperature are often performed at synchrotrons. Thanks to bright synchrotron radiation and modern fast large-area detectors, temperature-dependent diffraction has become one of the routine tools. However, designing an efficient heater to provide the temperature control is a challenge, especially for diffraction measurements where a large exit window is required to observe higher angle scattering.

Hot-air blowers are a common method for heating capillaries, but have some drawbacks in that they are often limited to low temperatures or low ramp rates, and large temperature gradients over millimeter-sized samples (Newton *et al.*, 2019 ▸). They are, however, versatile because the directed air flow allows for an open environment for a capillary. They have a long history of use in synchrotron experiments (van Beek & Pattison, 2019 ▸; Norby, 1997 ▸; Grubb *et al.*, 1984 ▸).

Resistive heaters are an attractive option. They provide heat through either conduction or radiation. Designs based around the use of ceramic elements surrounded by resistive wire have been used previously; the design by Chupas *et al.* (2008 ▸) is a well known example. This heater uses two ceramic pieces wrapped in kanthal wire above and below the capillary, and can reach 1000°C. One disadvantage of this design is the mechanical connection between capillary and heater which can make it difficult to align a sample. A later design incorporated a variable winding pitch in the resistive wire to give an intentional thermal gradient for measuring across a sample (O’Nolan *et al.*, 2020 ▸). A similar, more enclosed, design for small-angle measurements was reported by Andreasen *et al.* (2003 ▸). In this device the capillary is placed inside a ceramic tube wrapped in resistive wire. It also featured a vacuum chamber to reduce air scattering of X-rays. A high-pressure (1.5 kbar) hydro­thermal cell for extended X-ray absorption fine-structure spectroscopy (EXAFS) was reported by Hoffmann *et al.* (2000 ▸). The cell used 3.2 mm-diameter heat cartridges to provide temperatures up to 600°C.

Larger resistance furnaces have been used for X-ray diffraction experiments, notably Linkam furnaces used on a number of beamlines (Connor *et al.*, 2018 ▸). These are capable of reaching high temperatures (1500°C) and ramp rates (200°C min^−1^) (Oversluizen *et al.*, 1995 ▸; Bras & Ryan, 1998 ▸) and seem like a good option for solid state measurements, though the enclosed system may limit sample environment options. Mirror furnaces, in which light from a lamp is focused to a very small point, are another option for very high temperatures, although the temperature gradient is necessarily high and temperature control is notoriously difficult (Proffen *et al.*, 1995 ▸).

Doran *et al.* (2017 ▸) reported an infra-red tube furnace, in use at beamline 12.2.2 at the Advanced Light Source, capable of temperatures >1100°C. The design uses a SiC tube, in which a capillary is placed. The tube is heated by two infra-red lamps, which together run at 150 W with the furnace at 1200°C. The lamps are then surrounded by heat shielding. Two holes in the SiC tube act as an X-ray entry and exit window.

Energy-dispersive diffraction measurements also allow for a narrow exit window. Large reaction vessels with heating jackets were used in the early days of *in situ* crystallisation reactions in this way (Evans *et al.*, 1995 ▸; Barnes *et al.*, 1996 ▸).

Blichfeld *et al.* (2020 ▸) designed a resistive heater for thin film measurements capable of reaching temperatures of 1100°C at 1200°C min^−1^ using a small hot-plate made from Si_3_N_4_ by Bach Resistor Ceramics. The cell featured an aluminium casing and Kapton windows which allowed for gas flow to regulate the atmosphere. This was used to measure the *in situ* crystallisation of epitaxial or textured thin films from solution deposition methods.

Microwave devices have been reported in hydro­thermal reactors (Caffrey *et al.*, 1990 ▸). As microwave radiation directly heats the reaction medium, it is potentially more efficient than convection or heat radiative methods, and can achieve high ramp rates (Schmidt *et al.*, 2018 ▸). However, the application is limited to reactions in polar solvents.

Induction furnaces are used in high-temperature applications (Tang *et al.*, 1998 ▸; Kudrna Prašek *et al.*, 2018 ▸). They can be very efficient and reach temperatures >1500°C but have some disadvantages: it can be harder to control temperature if the sample itself is affected by the electric field, and they are more complex in design which can put constraints on sample environment.

There are, therefore, many options available for the heating of powder samples during diffraction or EXAFS experiments at synchrotrons that each have their advantages and dis­advantages. Modern synchrotron sources and detector systems allow for measurements of 1 s per diffraction pattern or even faster. In spite of the many solutions that are already reported and tested, there is still a need for a fast heater (*e.g.* 60–120°C min^−1^ temperature ramp rate that for 1 s exposure gives 1–2°C temperature sampling) that can reach temperatures above 1000°C and provides reliable and reproducible control of the temperature without a large gradient in the measured zone (that implies that the hot zone has to be larger than the zone irradiated by the beam). The heater has to be compatible with capillary sample holders, *in situ* and *operando* equipment, such as gas flow cells, and provide sufficiently large opening for both low-angle (small angles X-ray scattering, EXAFS) and high-angle (powder diffraction, pair distribution function) tools. Further design objectives comprise low thermal mass and a small axial thermal gradient especially important for *operando* space-resolved catalytic micro-flow reactors.

Here we report a design for a capillary heater which uses a SiC head piece and Si_3_N_4_ heat cartridges capable of reaching temperatures above 1000°C at a rate above 100°C min^−1^.

## 2. Description

**Figure 1:**
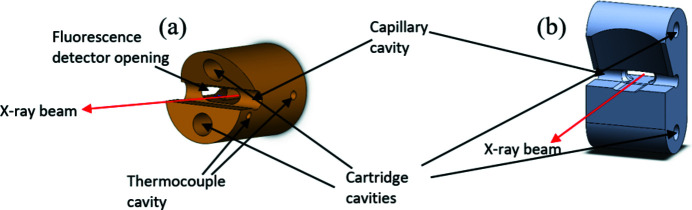
*SolidWorks* (https://www.solidworks.com/) drawings of the (*a*) in-line and (*b*) perpendicular SiC head pieces.

Two SiC head pieces with different designs were made: the ‘in-line’ piece and the ‘perpendicular’ piece, so called because of the relative directions of the heat cartridges with respect to the beam. These are each approximately 3 cm in their longest dimension. SiC was chosen due to its high thermal conductivity (>300 W m^−1^ K^−1^) (Yu & Levinshtein, 2001 ▸), stability and resistance to thermal shock. They each have opening angles of 60°, making them suitable for synchrotron X-ray diffraction (XRD) and total scattering experiments. Both have two holes cut out to fit 3 mm heat cartridges made from Si_3_N_4_ with coiled resistive wire running through. These are part 82306B by Webasto, normally used as a flame sensor in combustion heaters. The capillary can be placed in the cut between the cartridge holes. Smaller holes (1 mm) between the capillary and heat cartridges are cut to fit reference thermocouples. Fig. 1 ▸ shows 3D drawings of the SiC heads. The heater is controlled with a Eurotherm Nanodac, with power supplied using a Delta Elektronika box.

**Figure 2:**
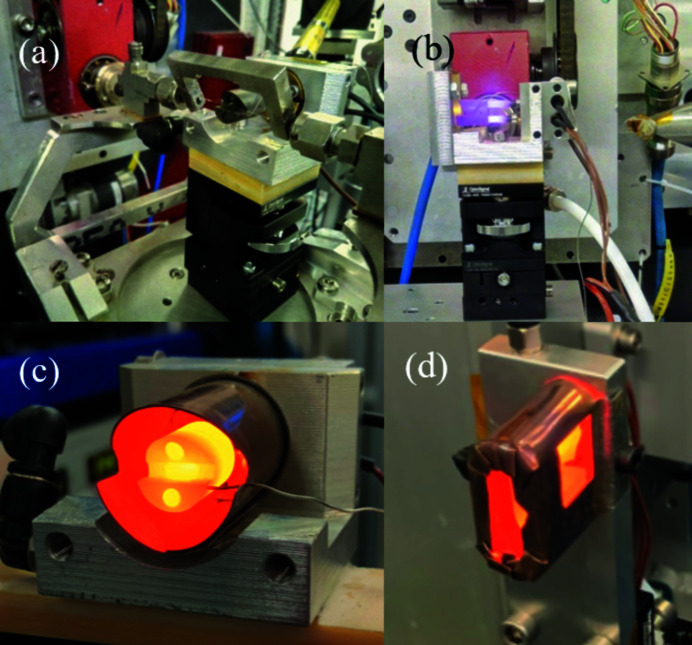
(*a*) Photograph of the in-line heater on BM31 (ESRF) with a gas flow bracket attached, (*b*) the heater during a test, and (*c*, *d*) the in-line and perpendicular heaters, respectively, with steel radiation shielding.

Fig. 2 ▸ shows photographs of the two heaters in their frames. Water-cooling channels flow through them, with 6 mm tube adapters at the ends. The heat cartridges are connected in parallel.

**Figure 3:**
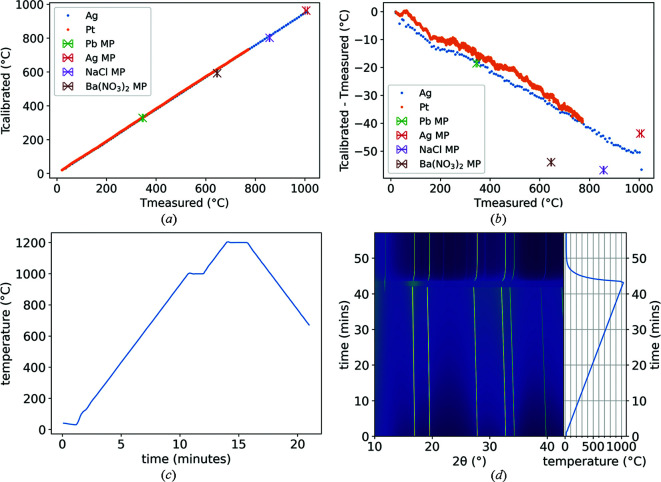
(*a*) The measured temperature (reference thermocouple) plotted against the calibrated temperature determined by lattice parameters (solid circles) and some melting points (X symbols). (*b*) The same plot as (*a*) but as a difference between measured and calibrated. (*c*) Logged thermocouple data of a ramp to 1000°C and then to 1200°C at 100°C min^−1^. (*d*) Contour plot of XRD patterns of Ag collected during a heating ramp to above its melting point (square root of intensity on colour-axis). Reference thermocouple temperature data are shown on the right-hand side of the plot. The phase which appears at around 40 min (941°C on thermocouple, 892°C calibrated, most prominent peak at ∼11.8°) is the quartz capillary crystallising at the elevated temperatures. Data collected at BM01, ESRF.

The heater has been tested and calibrated using lattice parameters, determined using X-ray diffraction of Ag and Pt, as well as with the melting points of Pb, Ba(NO_3_)_2_, NaCl and Ag up to 1200°C and 100°C min^−1^ [Fig. 3 ▸(*a*)]. A thermocouple log is shown in Fig. 3 ▸(*c*). These data were measured on the ‘in-line’ furnace. Lattice parameter calibrations were performed using a cubic relation; details are given in Appendix *A* [App appa]. The software *Tcal* was used to quickly calculate the calibrated temperatures from the diffraction data; details are given in Appendix *B* [App appb].

The heater is useful for measuring phase and lattice parameter changes due to its wide temperature range [*e.g.* the melting of silver (and crystallisation of quartz) shown in Fig. 3 ▸(*d*)]. It can also be used in conjunction with gas flow or gas isobar cells for measuring, for example, gas adsorption or absorption, or (catalytic) chemical reactions. Due to the low thermal mass and high shock resistance, the cell is capable of high heating and cooling rates which can be useful in kinetic studies. At maximum rate, the heater cools from 1000°C to room temperature in less than 10 min, saving experiment time and cooling time for changing samples.

**Figure 4:**
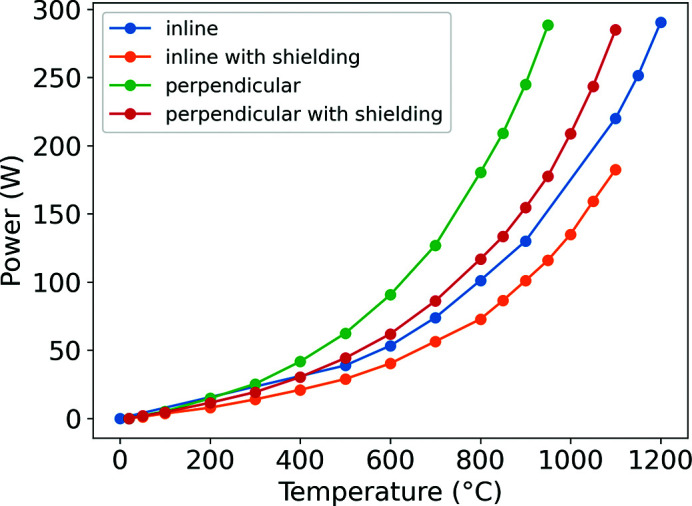
Plot of temperature against power consumption with the in-line and perpendicular designs, with and without radiation shielding.

Higher temperatures (>750°C) can only be reliably achieved when applying radiation shielding to the heater. Shielding substantially reduced power consumption and therefore improves the lifetime of the cartridges. At 1100°C, the in-line furnace uses 183 W and 220 W with and without shielding, respectively – a 17% reduction. The difference is starker with the perpendicular heater, with power being reduced from 288 W to 178 W at 950°C – a 38% reduction (Fig. 4 ▸).

**Figure 5:**
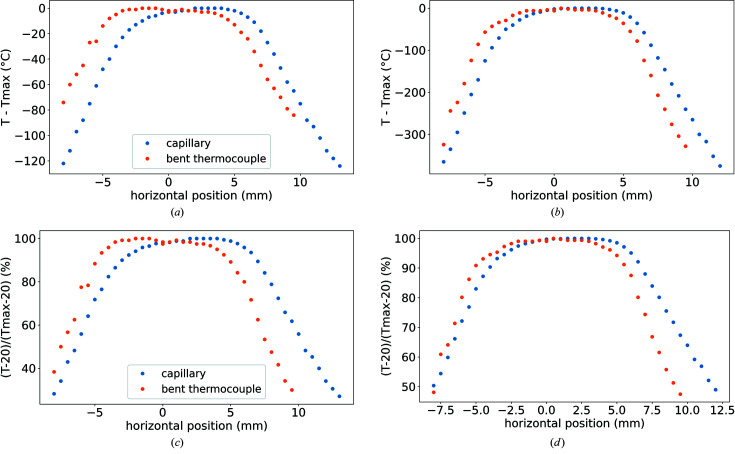
Axial temperature subtracted by maximum temperature measured at (*a*) 200°C and (*b*) 800°C, and relative temperatures at (*c*) 200°C and (*d*) 800°C measured using a thermocouple in two configurations: one in which the thermocouple was placed inside a capillary, and the other where most of the thermocouple was outside the heater with the tip bent into the heater’s mouth.

Fig. 5 ▸ shows the axial temperature distribution at 200°C and 800°C measured using two methods. The data for a thermocouple placed in a capillary are skewed such that higher temperatures are observed on one side of the heater than the other. This is the effect of thermal conduction in the thermocouple itself; when more of the thermocouple is in the heater, higher temperatures are observed. This experiment illustrates that a highly conducting sample will have a highly uniform temperature distribution. The second method used a thermocouple that was held mostly outside of the heater, with its tip in the capillary section to negate the effect of different temperature distributions within the thermocouple, resulting in less skew in the data. From these measurements we observe that the heater is within 1% of the maximum measured temperature within a 5 mm distance, both at 200°C and 800°C (using the bent thermocouple data); 2°C and 6°C, respectively. This is a substantial improvement on what we see from a hot-air blower which varies by 75% across 10 mm at the centre of the blower (Newton *et al.*, 2019 ▸).

For capillary measurements we consider this 5 mm window of 1% variation to be a low gradient. Since the heater can be kept consistently in the same position with respect to the beam (we estimate this to be well within 1 mm), the sample-to-sample temperature variation is negligible, and will depend more on the sample’s thermal conductivity, quantity and how symmetrically it is placed in the heater.

## 3. Conclusion

We have designed and tested a new resistive heater for use on synchrotron beamlines. It is useful for high-temperature measurements (>1000°C) and ramp rates (>100°C min^−1^) using X-ray diffraction and X-ray absorption spectroscopy, and compatible with gas flow and pressure cells because of its open design and compact size. The device is ideal for measuring capillary samples in a variety of environments to study, for example, phase change, chemical reactions including under solid-state, solvothermal and catalytic gas flow conditions, gas isotherms or isobars.

## Figures and Tables

**Table 1:** Coefficients for the calibration function refined from thermal expansion data from Kirby *et al.* (1977 ▸) *T*_max_ refers to the maximum measured temperature in the data.

Material	*b*_0_ (K)	*b*_1_	*b*_2_	*b*_3_	*T*_max_ (K)
Ag	293.21939	5.216 (7) × 10^4^	−4.6 (1) × 10^5^	1.0 (4) × 10^6^	1200
Pt	293	1.1101 (7) × 10^5^	−1.43 (1) × 10^6^	7.4 (6) × 10^6^	1800
Si†	293	3.47 (5) × 10^5^	−4.6 (5) × 10^7^	6(1) × 10^9^	1200

† Silicon has a much smaller thermal expansion than Ag or Pt (about six times less and three times less, respectively), and so is less reliable.

## Appendix A

### A1. Calculation of temperature from thermal expansion

The thermal expansion is defined as

where *a* is a unit-cell dimension of the reference material measured as a function of temperature, and *a*_20_ is the value taken at 20°C.

Temperature dependence of the thermal expansion can be approximated as a polynomial function,

The temperature at the sample position is calculated from the thermal expansion of a reference material placed at the same position as the sample. For calibration purposes it is convenient to use an inverted function,

where coefficients *b*_*i*_ are refined from the tabulated data on thermal expansion of the reference materials (Ag, Pt) and given in Table 1 ▸.

### A2. A note on the accuracy of temperature calibration from thermal expansion

We first assume the same resolution *R* =  ≃ 10^−4^ for all datasets.

Error propagation [equation (1)] gives

with a linear coefficient of thermal expansion

and, with the prior assumption, one obtains

For silver at *T* = 1200°C, α = 29.6 × 10^−4^ K^−1^ and ɛ = 0.21180, this gives δ*T* = 0.05 K. This estimate has to be taken as a lower limit for the calibration based on thermal expansion, as it neglects uncertainties for ɛ and α, as well as errors coming from the experimental instabilities.

## Appendix B Tcal

We have developed software, *Tcal*, for performing fast temperature calibration using the thermal expansions of silver or platinum in use at BM01, and integrated into the *Pylatus* software to apply calibrations during measurements (Dyadkin *et al.*, 2016 ▸). It finds positions of diffraction peaks to determine the lattice parameter of the material, and compares this with the value at room temperature, *i.e.* zero power through the heating device in use (the calibration value is assumed to be equal to the measured temperature at this point). *a*_0_ of the calibrant is refined against the room-temperature value, or it can be fixed if desired, and used in equation (1) to determine the temperature of the calibrant in each measured diffraction pattern. The software can read file headers in the .cbf images to find the wavelength if available, otherwise it can be entered as an argument. The source code (written in *Rust*) is available at https://git.3lp.cx/dyadkin/tcal and is freely available to be downloaded and compiled.
